# Assessment of varying changes of vegetation and the response to climatic factors using GIMMS NDVI3g on the Tibetan Plateau

**DOI:** 10.1371/journal.pone.0234848

**Published:** 2020-06-17

**Authors:** Yuke Zhou, Junfu Fan, Xiaoying Wang

**Affiliations:** 1 Key Laboratory of Ecosystem Network Observation and Modeling, Institute of Geographic Sciences and Natural Resources Research, Chinese Academy of Sciences, Beijing, China; 2 Southern Marine Science and Engineering Guangdong Laboratory (Guangzhou), Guangzhou, China; 3 School of Civil and Architectural Engineering, Shandong University of Technology, Zibo, China; 4 Institute of Atmospheric Environment, CMA, Shenyang, China; Irstea, FRANCE

## Abstract

Under the context of global climate change, vegetation on the Tibetan Plateau (TP) has experienced significant changes during the past three decades. In this study, the spatiotemporal changes of growing season vegetation index (GSVI) on the TP were analyzed using various methods from pixel level to ecoregion level. In addition, a relative importance approach was employed to investigate the regulating effect of temperature and precipitation on vegetation. During the period of 1982–2012, vegetation on the TP was generally experiencing a greening trend, but with pronounced fluctuations. The interannual variation of the long-term GSVI was most significant in the Qaidam Basin and southern forest. At ecoregion scale, vegetation in the arid and frigid arid zones showed a browning tendency, with other ecoregions presenting greener trends. Over a large proportion of the TP, there exist change points in the GSVI time series, which were mainly concentered around the year 1996 and 2000. The Hurst exponent identified that a majority (88%) of the vegetation on the plateau would maintain a persistent trend in the future, which would mainly consist of undetermined development and greening trends. TP vegetation during the 1990s experienced more greening than in the 1980s or 2000s according to the interdecadal analysis. The long-term change in growing season vegetation was most positively correlated with the temperature during the same period, followed by the temperature in the preseason and postseason periods. There were more negative relationships of vegetation change with precipitation than with temperature. The relative contribution of the temperature to the vegetation changes exhibited an opposite spatial pattern to that of precipitation. Overall, the findings in this work provide an essential archive of decade-scale vegetation dynamics that may be helpful for projecting the future ecosystem dynamics on the Tibetan Plateau, such as the consistent greening.

## Introduction

Vegetation plays a pivotal role in land surface processes by affecting the regional climate, the water and carbon cycle, soil moisture, etc.; in turn, vegetation responds to global changes [1–3]. Previous studies have widely reported that global climate change has essential impacts on the biosphere [4,5]. In the northern mid- and high-latitude regions, apparent surface air temperature warming has substantially promoted vegetation production by prolonging the growing season length and photosynthetic activity [6,7]. In China, land surface vegetation has also experienced a pronounced greening trend under the drivers of climate change and human management activities [8]. At the regional scale, the vegetation change in China was also identified to be related to climate warming, especially on the Tibetan Plateau (TP) [8]. In the study domain, the TP is commonly considered an ideal study area for verifying climate change and ecosystem responses due to its fragile environment [9–12]. A considerable number of studies have focused on the vegetation dynamics on the TP, which commonly detected linear trends in the vegetation growth or its correlation to environmental factors [13,14]. Thus it is of great importance to comprehensively analysis the spatiotemporal pattern of vegetation dynamics over the TP.

Remotely sensed NDVI (i.e., normalized difference vegetation index) is an extensively used greenness index that is a suitable proxy for plant photosynthesis and vigor [15,16]. The availability of various satellite remotely sensed NDVI (e.g., GIMMS NDVI3g, Spot VGT and MODIS NDVI) enabled us to obtain more information about vegetation changes and the climatic causes from a long-term and global perspective [17,18]. Previous studies have revealed that vegetation has presented a significant spatiotemporal variation due to location-specific environmental characteristics [8,19]. On the TP, vegetation changes have shown a complex pattern in different vegetation types [9,20,21]. Studies have reported that natural grasslands have been degrading since the 1980s, which may be due to a combination of climate change and anthropogenic activities (e.g., increasing population and overgrazing) [22–24]. Current literature has demonstrated that the TP has converted from a small carbon source to a carbon sink during the 20th century, and the simulated net primary production (NPP) will persistently increase over the last 50 years [20,25]. A model-based study also identified a greening trend in the vegetation throughout the TP under various climate change scenarios [21]. With the melting ice and rising temperature during the warm season, vegetation is growing more rapidly, resulting in a larger land cover range and a strong peak during the growing season throughout the entire TP [26]. The increase in NDVI during the growing season could be partly attributed to the earlier onset of the growing season and accelerated vegetation activity [27]. Generally, the majority of these studies have applied the slope of a linear regression to depict the vegetation development trends. In addition, few studies have examined the consistency of vegetation dynamic trends after their study periods based on satellite remotely sensed NDVI. Thus, more suitable methods should be applied to detect the vegetation dynamics by combining the spatial and temporal information.

Due to the high altitude and cold environment, the TP alpine vegetation (mainly grassland) is sensitive to climate warming and human activities [28,29], making it an ideal place for exploring the correlation between the effects of climate warming and anthropogenic activity on alpine vegetation. For the TP, increasing temperatures commonly seem to be the major forces driving vegetation greening [13,30,31]. One study documented that a major climatic factor limiting the production of the grassland ecosystem was the low thermal conditions on the TP [24]; thus, an increase in temperature may increase the productivity of the grasslands on the plateau [14]. The increased growing season vegetation activity throughout the TP may in turn slow surface warming through enhanced evapotranspiration [32]. Among the previous discussions on the relationship between climatic factors and vegetation on the TP, they were mainly focused on the response of vegetation to environmental factors in the same time windows, such as annual NDVI to annual temperature and the growing season NDVI to the corresponding temperature. The time-lag effect, in which the responses of vegetation to climate have a certain time lag, [33] has not been widely examined on the TP. In the case of a time-lag effect, the preseason climatic conditions would contribute to the vegetation development status during the growing season. In this study, the relationship of temperature and precipitation with the NDVI during the preseason, growing season and postseason were investigated.

In summary, the long-term trends and interannual variability of vegetation and the corresponding climatic drivers throughout the TP remain poorly understood. The main objectives of this study were to (1) examine the growing season NDVI trends and variations across multiple spatiotemporal scales using the Theil-Sen slope, EOF (empirical orthogonal function) and Hovmöller diagrams; (2) detect the change points and spatial pattern in the long-term NDVI sequence at the pixel level; and (3) explore the relative contributions of temperature and precipitation in affecting the growing season vegetation.

## Materials and methods

### Study area

**Fig 1:**
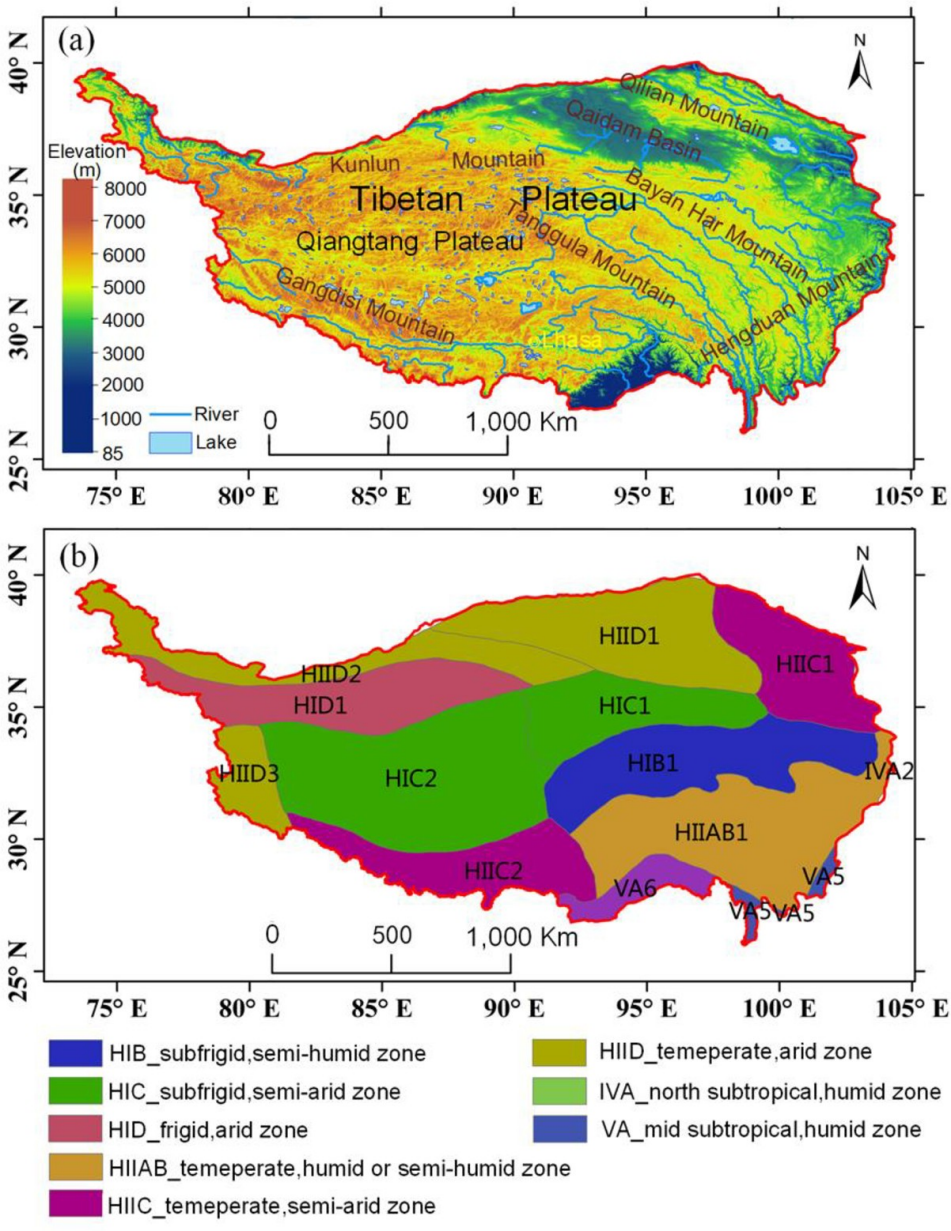
Map of terrain (a) and ecoregions (b) of the Tibetan Plateau.

This paper focused on the study area of the TP due to the sensitive response of vegetation to climate change and its ecological vulnerability [28,29]. The Tibetan Plateau, which is located in southwestern China, encompasses an extent of 73°18’-104°46’ E and 26°00’-39°46’ N, covering approximately 2.57 × 10^6^ km^2^. Known as the “roof of the world” [34,35], with an average elevation higher than 4000 m above sea level, its topographic pattern shows an increasing altitudinal gradient from the Qaidam basin in the northeast to the Qiangtang Plateau, Kunlun Mountains and Gangdis Mountain in the west (Fig 1A). The typical vegetation types on the TP mainly consist of grass, forest and shrub. Grass is the dominant plant, including alpine steppe and meadow, and is mostly distributed in the central region. The forests and shrubland mainly grow in the southeast and occupy a small proportion of the vegetation throughout the plateau.

In this study, the ecoregion scale was also considered for the detecting of trends and interannual variability of vegetation, which is a medium spatial scale between the entire plateau and pixel scales. The ecoregions were planned considering not only the climatic factors, such as temperature and precipitation, but also including vegetation information, such as plant types and covering condition [36]. While the natural distribution of vegetation is governed by climatic factors, the vegetation may present a significant homogeneity with these factors [36,37]. Thus, exploring the long-term trends and variations in vegetation in these ecoregions will improve our understanding of vegetation evolution and identify the differences between ecoregions. The TP was mainly divided into eight ecoregions, including four key temperature zones (the highland temperate, frigid, subfrigid and subtropical zones) and five humid-arid zones (the semihumid, semiarid, arid, humid or semihumid, and humid zones) (Fig 1B) [38].

### GIMMS NDVI3g data and preprocessing

In this study, the third generation GIMMS NDVI (NDVI3g) dataset was used to analyze the vegetation dynamics and trends over the TP (obtained from http://ecocast.arc.nasa.gov). This dataset was derived from Advanced Very High Resolution Radiometer (AVHRR) instruments and covers the time range from 1981–2012 with a spatial resolution of 8 km and temporal resolution of 15 days [39]. During the data assembly process, the data quality of this product was calibrated by correcting for the effects of the solar angle, errors in the different AVHRR sensors, cloud cover and volcanic eruptions. To date, this is the longest time series of NDVI data and is suitable to capture the long-term trends of vegetation activity [40]. For regional specificity, NDVI3g is a reliable data product widely used in ecosystem studies of arid and semiarid regions [40,41].

We extracted the NDVI and the corresponding quality FLAG layers from the GIMMS NDVI3g product and then converted them from the VI3g format to the GIS friendly GeoTiff format. After format conversion, these raster files were clipped to the geographic range of the Tibetan Plateau. For each pixel, the quality of the NDVI value was controlled with the FLAG reliability indicator (FLAG = 1, 2, 3, 5). Thus, the quality-checked NDVI files were built into a raster stack according to their temporal order using the R package ‘raster’ [42]. In the raster stack, the time series for each pixel was smoothed using the Savitzky-Golay filter with a moving average window of seven points, which was useful for reducing short-term NDVI signal distortion and noise. The NDVI time series were subsequently replaced with smoothed values. Pixels with annual mean NDVI < 0.05 that commonly refers to barren areas, were excluded from trend and variation analyses.

Because of the high photosynthetic activity of the vegetation and their carbon exchange with the ecosystems during the growing season, we took the annual growing season NDVI (GSVI) value as an indicator of the comprehensive vegetation growth conditions for the whole year. Hereafter, without specific explanation, the GSVI refers to the averaged NDVIs of the growing season months, which could avoid the effects of abnormal values outside the growing season. Following previous studies, growing season in the TP was defined as May-September [32,43], which covers a period that includes ten semimonthly composited satellite images of the NDVI. In this case, the pre- and postseason were defined as January-April and October-December, respectively, in the current year.

### Climatic dataset

**Fig 2:**
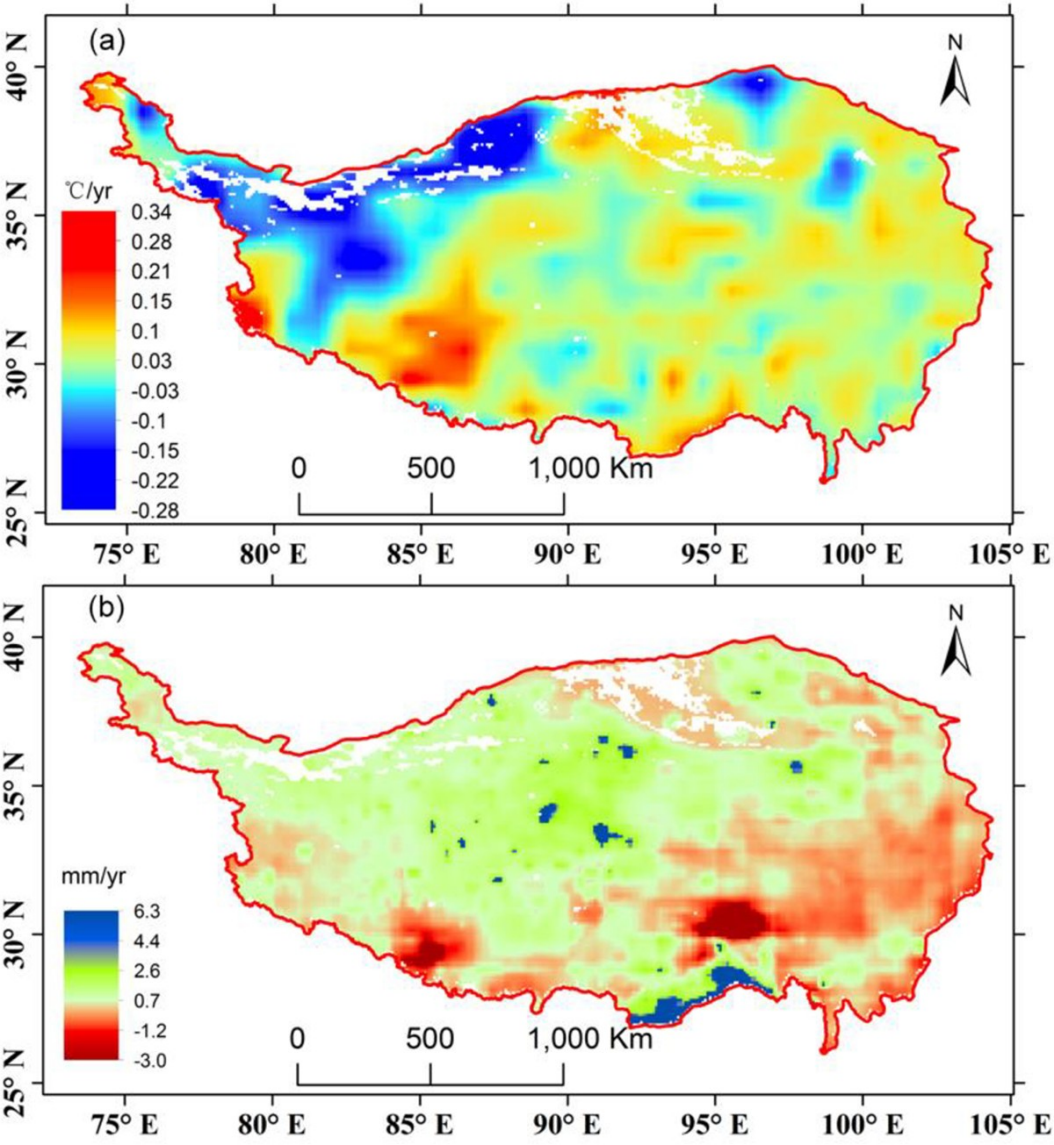
Long-term trends of monthly temperature (a) and precipitation (b) on the TP.

For this study, the temperature and precipitation raster data were obtained from the China Meteorological Forcing Dataset, which was produced by merging a variety of data sources [44,45]. The data sources used to produce the forcing data include the CMA (China Meteorological Administration) station data, TRMM satellite precipitation analysis data, Princeton forcing data, and GLDAS data. This dataset has spatial and temporal resolutions of 0.1 degrees and 3 hours, respectively. To use in combination = with the GIMMS NDVI3g data, these data were resampled to 8 km using the nearest neighbor method and aggregated into monthly data (Fig 2).

### Trend and variation analysis

#### Theil-Sen trend estimation

It has been a widely discussed and complex research question to determine whether the terrestrial vegetation throughout the TP is greening or browning [21,46]. We aimed to determine whether the NDVI values generally increased or decreased over the past three decades. Theil-Sen trend estimation and the Mann-Kendall test were employed to investigate the long-term trend of the vegetation on the TP. We used the Theil-Sen method to estimate the slope of the trend for each pixel. The Theil-Sen estimator, named after Henri Theil and Pranab Sen, was an efficient linear trend detection method. The Theil-Sen estimator is not sensitive to outliers in time series and has been used prevalently in astronomy and environmental studies [47–49]. The magnitude of the slope can be computed as seen in Eq 1 [50].

$$b_{sen}=Median\left[\frac{Y_{i}-Y_{j}}{\left(i-j\right)}\right]\;for\;all\;i>j$$ (1)

where *Y*_i_ and *Y*_j_ are the observed data at corresponding time points i and j. If the time series totally consists of n observations, then there will be (n(n-1))/2 estimated slopes. *b*_sen_, the result of the statistical test, is derived from the median of all estimated slopes.

The Mann-Kendall test, which is a nonparametric test suitable for handling nonnormally distributed data, has been widely used to depict trends in climatic and hydrologic time-series data [51,52]. This test is not sensitive to interference from a few outliers and is particularly effective for short-term time-series data [53]. In this study, we applied the p-value calculated from the Kendall R package [54] to determine the significance level of the Theil-Sen trend.

#### Coefficient of variation

The relationship between the mean and dispersion is very important in the geosciences and is expressed by the coefficient of variation (CV) [55]. The CV represents the ratio of the standard deviation to the average value [56] and is commonly expressed as a percentage (Eq 2).

CV%=100σ/mean (2)

where σ is the standard deviation.

#### Hurst exponent and detection of future vegetation trends

While trend detection is widely used in the study of long-term vegetation dynamics, it is worth exploring whether the current plant development tendencies will be sustained in the future. Here, the Hurst exponent was applied to detect the future tendency of the vegetation on the TP. In detail, the rescaled range (R/S) method based on the Hurst exponent (H) was chosen to measure the long-term memory in the GSVI time series [57]. The R/S analysis was used to estimate the autocorrelation properties of the time series [58]. A time series of length N was divided into a number of shorter time series of length *n = N*, *N/2*, *N/4*,…; the average rescaled range was then calculated for each value of n as follows [59]:

1. As *X = X*_*1*_, *X*_*2*_, …, *X*_*n*_, calculate the mean value m as follows:
  $$m=\frac{1}{n}\sum_{i=1}^{n}X_{i}$$ (3)
2. Derive a new time series adjusted by m.
  $$Y_{t}=X_{t}–m,\;t=1,2,\ldots ,n$$ (4)
3. Calculate the cumulative deviation series Z as follows:
  $$Z_{t}=\overset{t}{\underset{i=1}{\sum }}Y_{i}$$ (5)
4. Compute the range R as follows:
  $$R\left(n\right)=max\left(Z_{1},Z_{2},\ldots ,Z_{n}\right)‑min\left(Z_{1},Z_{2},\ldots ,Z_{n}\right)$$ (6)
5. Compute the standard deviation S with the following formula:
  $$S(n)=\sqrt{\frac{1}{n}\sum_{i=1}^{n}{\left(X_{i}-m\right)}^{2}}$$ (7)
6. Calculate the rescaled range *R(n)/S(n)* and average it over all the partial time series of length n. The H value is derived by fitting the following formula:
  $$\frac{R(n)}{S(n)}=Cn^{H}$$ (8)

The *H* value varies from 0 to 1. The values 0.5 < *H* < 1 indicate persistence in a time series. The persistent features in a time series will become more significant as the *H* value approaches 1. An *H* value below 0.5 indicates a time series with anti-persistent properties, indicating the developing trend of the time series may change direction in the future. In this study, the *H* value was computed at the pixel level to detect the persistence of the GSVI time series.

To characterize various future development patterns of the vegetation on the TP, we used Theil-Sen trend slopes and the Hurst exponent together to classify the potential future trends into six types (Table 1). If a pixel had a positive slope and its *H* value was above 0.5, the vegetation in this pixel would tend to maintain a positive development (PD) status after 2012. When the slope was positive but its *H* value was smaller than 0.5, the vegetation in this pixel would maintain anti-persistent positive development (APD) after 2012. If the slope was negative and its *H* value was larger than 0.5, we classified its vegetation as having a negative persistent development (ND) in the future. When the *H* value was less than 0.5, it indicated anti-persistent development (AND). When a pixel had a nonsignificant Theil-Sen slope and an *H* value above 0.5, the vegetation in this pixel was supposed to maintain sustained and steady development (SSD). If the *H* value was less than 0.5, the vegetation was categorized into the type of undetermined development (UD).

**Table 1:** Classifications of the persistence in vegetation development.

	Persistent (0.5 < *H* < 1)	Anti-Persistent (0 < *H* < 0.5)
**Increasing**	Positive Development (PD)	Anti-persistent Positive Development (APD)
**Stable**	Sustained and Steady Development (SSD)	Undetermined Development (UD)
**Decreasing**	Negative Development (ND)	Anti-persistent Negative Development (AND)

#### Change point detection

In addition to the linearly developing trend of the time series, the change point is an important indicator of the variability of the vegetation on the Tibetan Plateau. Vegetation may have various growing patterns in the long term and will not always follow a gradual change pattern. The analysis of change points helps to gain insight into the extremes of the vegetation conditions at the regional scale, such as wildfires, harvesting or diseases. Generally, change points can reflect abrupt browning or greening conditions of plants induced by changes in climatic factors, such as temperature, precipitation, and solar radiation [60,61].

There are a variety of methods for detecting change points in time-series data, such as the Pettitt test [62], segmented linear regression, standard normal homogeneity test (SNH) for a single break [63], BFAST [64] and Buishand range test [61]. Here, we employed the widely used Pettitt test method to find the most significant change point for each pixel during the period from 1982–2012. The Pettitt test is a nonparametric method for detecting change points in time-series observation data. Because the Pettitt test requires no assumption about the distribution of the data, it has been commonly used in climatic and hydrologic studies for a long time. In the Pettitt test, the null hypothesis, H0, is that the data in the time series are independent and randomly distributed, while the alternative hypothesis, H1, is that there is a time point t that divides the time series into two parts that follow different distributions. In other words, the Pettitt test assumes that there is no change point in the interannual growing season NDVI process. Alternatively, the located change point t indicates that there was a point of sudden, rapid change in the time series. We took the following steps to detect change points in the GSVI time series:

1. Rank the annual GSVI data (X) from 1 to *N* (i.e., *X*_*1*_, *X*_*2*_,…,*X*_*n*_).
2. Calculate the value of *Vi* as follows:
  $$V_{i}=N+1-2R_{i},\;i=1,2,\cdots ,N$$
  where *R*_*i*_ is the rank of *X*_*i*_ in the GSVI time series.
3. Calculate the value of *U*i as follows:
  $$U_{i}=U_{i‐1}+V_{i}$$
  $$U_{1}=V_{1}$$
4. The most likely change point occurred at the *i*th observation when the following was true:
  $$K_{N}=max_{1<i<N}\left|U_{i}\right|$$
5. Estimate the critical value of *P*_oa_.
  $${P_{OA}=2e}^{\frac{6{K_{N}}^{2}}{-(N^{3}+N^{2})}}$$

If *P*_oa_ <*α*, where *α* is the statistical significance of the test, then the null hypothesis was rejected. In this study, *α* was set as 0.05.

All the satellite images of the annual growing season NDVI were integrated into a time series raster stack from 1982 to 2012 using the R package ‘raster’ [42]. Each pixel located in the study region was subjected to the Pettitt test to investigate the presence of a change point under the 0.05 significance level. The output image had the same geographical coordinate information and stored the change time (year) value using the integer type. Pixels with nonsignificant change points were set as having no data.

#### Empirical orthogonal function-based spatiotemporal analysis

We employed empirical orthogonal function (EOF) analysis and the temporal feature space to characterize the variation pattern of the growing season vegetation on the TP. The EOF analysis is commonly used to decompose multivariate datasets in terms of the orthogonal basis functions, which refers to the same set of principal component analysis (PCA) procedures [65,66]. EOF analysis denotes an approach for decomposing spatiotemporal fields into a set of independent orthogonal patterns. In meteorology and climate research, this approach enhances the description of regions that exhibit above average capabilities to explain the variance over time in a specific spatial domain [66]. Temporal EOF analysis provides a statistical basis for representing the spatially sufficient temporal patterns in the image time series as uncorrelated vectors of the GSVI value as a function of time from 1982 to 2012. The output EOF raster maps were listed in decreasing order of variance. This enabled a reduction in the number of output maps because the last of the transformed maps had little or no variation (may have been virtually constant maps). After several adjustments of the mode number parameter, the first ten leading modes were chosen because they explained approximately 99.5% of the original GSVI. The last components thus did not add significance and were discarded. The detailed effect of the EOF application on GSVI will be demonstrated in the results section.

#### Correlation and relative contribution of the climatic factors and GSVI

Climate conditions have significant effect on vegetation growth in terrestrial ecosystems. Detecting the relationship between the vegetation and climatic factors has been widely performed in vegetation dynamics studies [26,27]. Vegetation growing state is affected not only by the environmental variables during the growing season but also by these variables during the preseason period, which is known as the time-lagged effect on the climate-vegetation interaction [33]. Meanwhile, vegetation growth state will impact local climatic conditions in the postseason as a feedback to climatic change [67,68]. Thus, the temperature and precipitation in one year were divided into three time periods: preseason (Jan-Apr), growing season (May-Sep), and postseason (Oct-Dec). The mean temperatures in the three time periods were chosen and defined as PreT, GST, and PostT, while the summary of precipitation in the three time periods were defined as PreP, GSP, and PostP. Here, we investigated the correlations of GSVI with temperature and precipitation in the three time periods using the Spearman correlation coefficient (*r*).

Additionally, relative contribution analysis was performed to statistically analyze the roles of different climatic factors playing on vegetation responses. Here, we used the Lindeman-Merenda-Gold (LMG) method, which enabled us to determine the contribution of different correlated repressors into a multiple linear regression model. First, a multiple linear regression model was established in which GSVI was the dependent variable and temperature and precipitation were the independent variables. Then, we decomposed the linear model explained variance into the nonnegative contributions of temperature and precipitation to the GSVI [69,70]. The sum of the two relative contributions equaled the R2 derived from the linear regression. To better present the result, we standardized the data of the two relative contributions so that they sum to 100%. The R package relaimpo was used to compute the relative importances of the climatic factors [71]

In this study, the data processing and statistical analysis were performed in the R scientific computation environment (version 3.4.1, R Core Team, 2017) and the associated packages contributed by the user community, such as raster, rasterVis, zyp, sp, and ggplot2 (http://cran.r-project.org) [72].

## Results

### Vegetation spatial pattern and temporal dynamics

**Fig 3:**
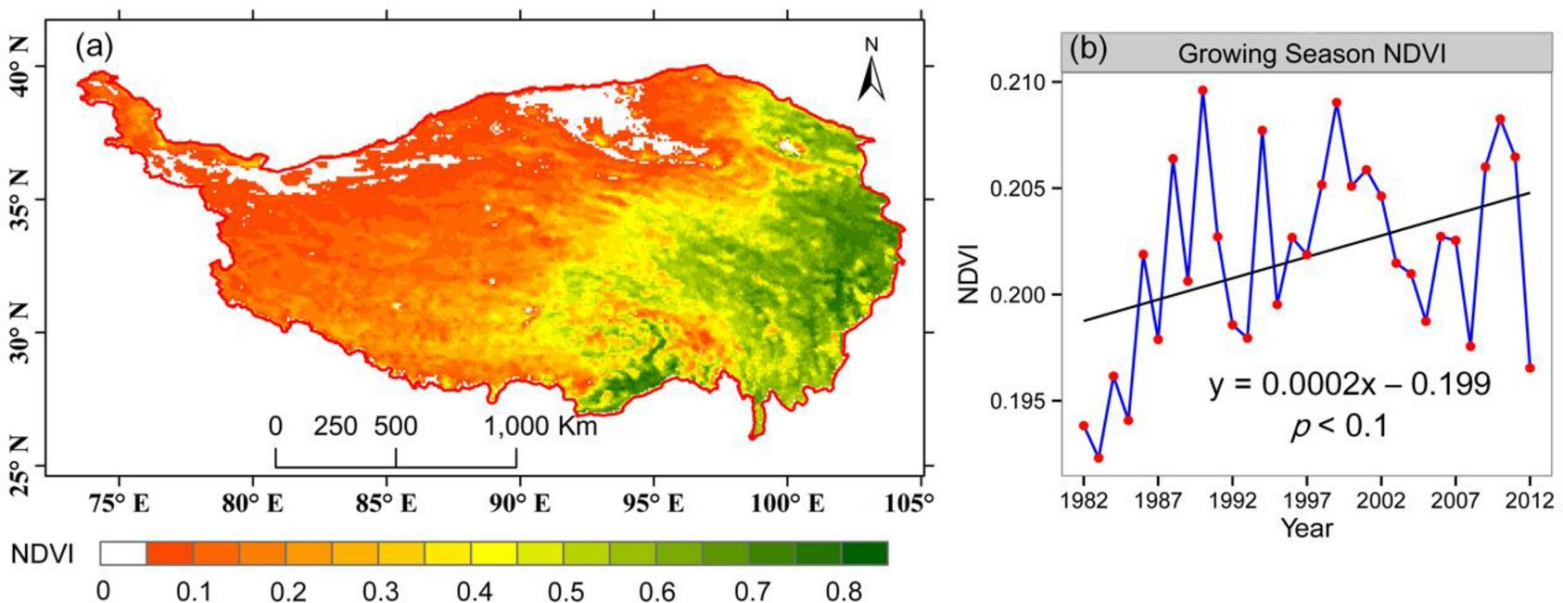
Spatial distribution of the 31-year averaged GSVI (a) and the time series of GSVI (b) on the entire TP during 1982–2012.

As shown in Fig 3B, for the entire region, the maximum GSVI occurred in 1990, while the change point was 1987 (at the significance level of 0.05 in the Pettitt test), while the linear trend is not significant (0.05< *p* < 0.1). In the spatial pattern, the multiyear averaged GSVI showed a decreasing trend from the southeastern to northwestern TP, which was consistent with the distribution of the climatic factors and terrestrial characteristics. According to the simple linear regression for the long-term GSVI, there was a slightly increasing trend in the GSVI with a slope of 0.0002 for the entire study period. There were two clearly distinct periods with opposite trends before and after 1997. The period before 1990 exhibits a greening trend of vegetation despite the abrupt decrease in 1987, which was followed by a decreasing trend until 1995. Then, the GSVI during the period from 1995–2000 reversed to showed an increasing trend. The decreasing trend in GSVI between 2000 and 2012 was partly in agreement with a previous study, which may be attributed to the declining temperature trend during the growing season. [6,73]

**Fig 4:**
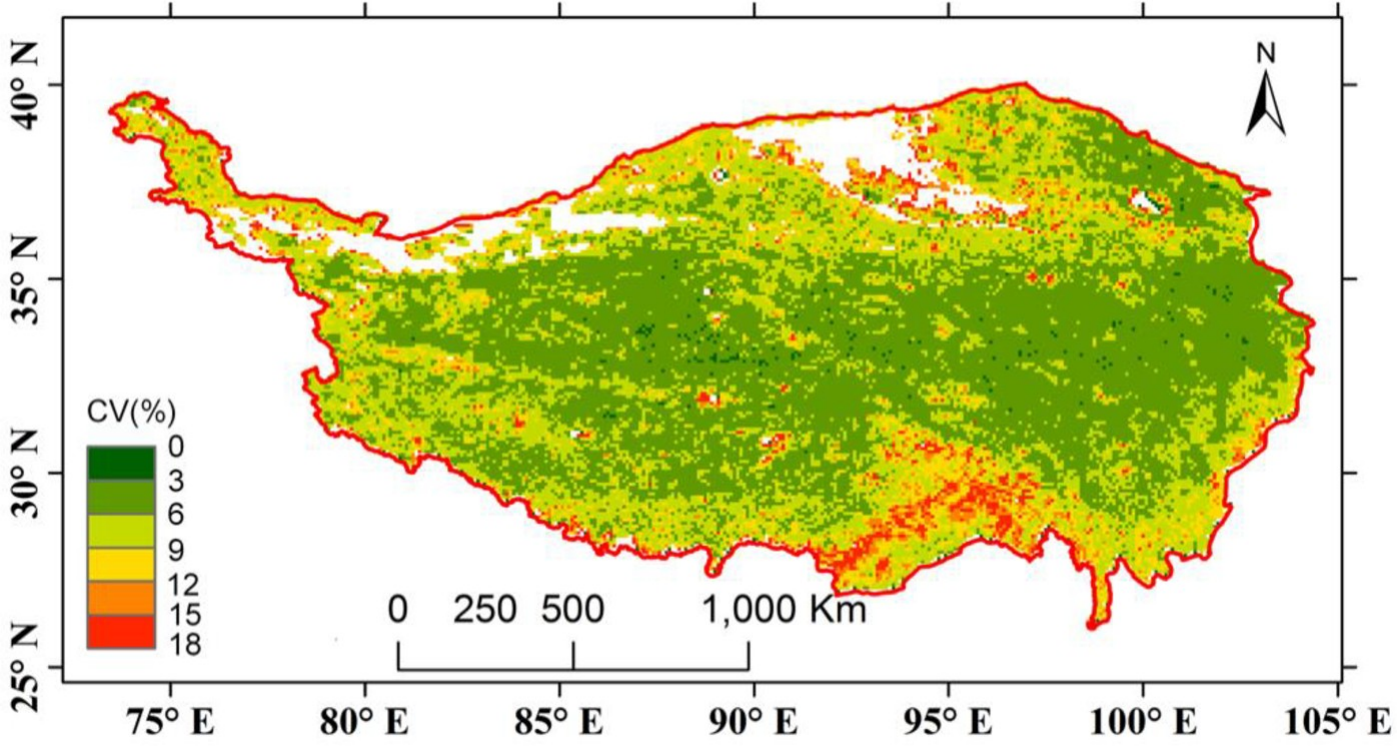
Spatial pattern of the coefficients of variations (CV) of the GSVI on the TP (1982–2012). Green color indicates small variation, while red color indicate high variation. The unit of CV is percentage (%).

The interannual variation in GSVI was investigated using the coefficient of variation (CV) at the pixel level (Fig 4). Significant variation emerged in the southern forest and marginal zone around the Qaidam basin. The majority of the vegetation on the TP did not exhibit prominent variation from 1982–2012.

### Differences in the GSVI between three decades

**Fig 5:**
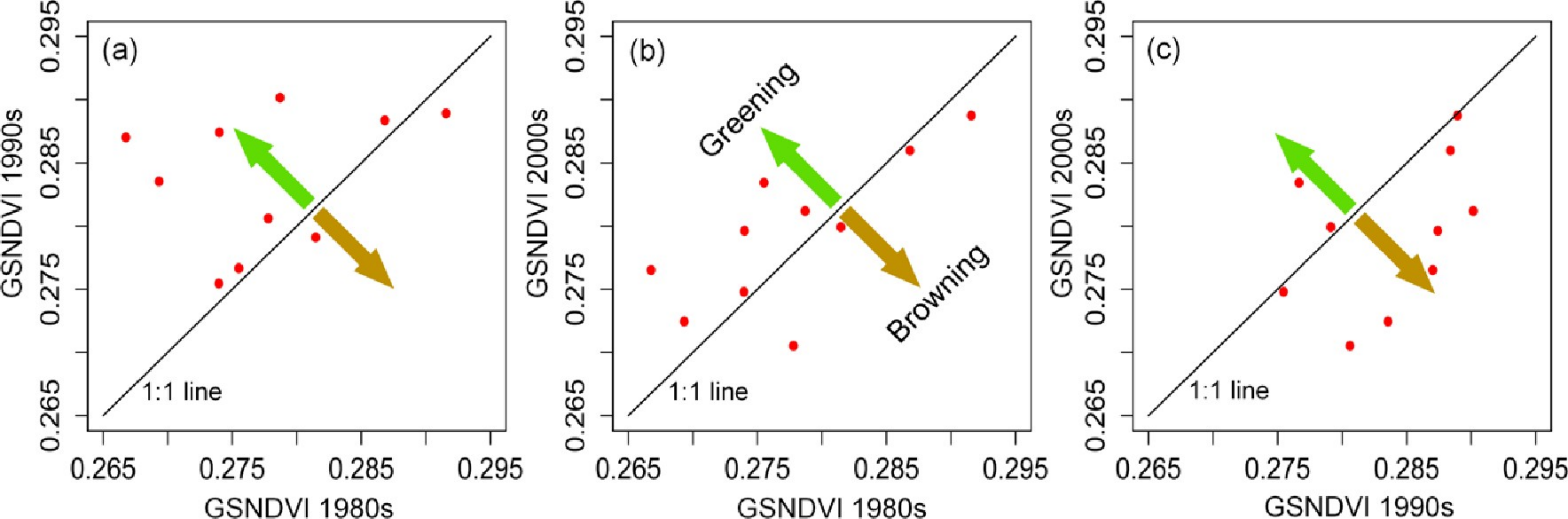
Comparison of the GSVI (red points indicate the annual GSVI) between the 1980s, 1990s and 2000s. The x-axis and y-axis represent GSVI in the former and later decade, respectively. The green and brown arrows separately demonstrate the greening and browning trends of the TP vegetation.

To characterize the vegetation variation of the interdecadal time periods, we separated the range of the study period into three selected 10-year epochs (1982–1991; 1992–2001; and 2002–2011), which approximately represent the time windows of the 1980s, 1990s and 2000s. The GSVI values through the TP in the three decades were compared to each other by drawing scatter plots (Fig 5). We set the GSVI in the former decades as the X axis and those in later decades as the Y axis, so the upper-left part of the scatterplot indicates a greening tendency of vegetation, while the lower-right part shows a browning trend. A point located near the 1:1 line means that there was no apparent change in the vegetation for the two corresponding time points in different decades. In Fig 5A, most of the points are located above the diagonal line with the exception of two points that are under that line. Additionally, six points are located near the diagonal line, but there are four points located in the upper part that are far away from the diagonal line. This result indicates that the vegetation from the 1980s to 1990s experienced a greening trend. In the comparison between the 1980s and 2000s, we found that there were nearly the same numbers of points on either side of the diagonal line, indicating that were was no significant change trend between the 1980s and 2000s (Fig 5B). In Fig 5C, most of the points are under the diagonal line, demonstrating the browning trend of the vegetation from the 1990s to 2000s.

According to the mean GSVI for the three time intervals (0.2776, 0.2837 and 0.2793), this also reflected that the vegetation activity in the 1990s was more intensive than that in other decades. This finding was consistent with the study results from Xu W et al 2007 [31]. From Fig 3B, this situation can be found by the convex fluctuation curve in the 1990s (especially from 1996–2000). A Welch t-test was applied to the GSVIs of the three decades to detect the differences between them. The t-test between the 1980s and 1990s yielded a t-value of -2.090 and a p-value of 0.052. That between the 1980s and 2000s obtained a t-value of -0.575 and a p-value of 0.573 and that between the 1990s and 2000s obtained a t-value of 1.749 and a p-value of 0.097. The mean GSVI of the 1990s was significantly different from that of the 1980s and 2000s. These results tend to indicated that the 1990s was a unique epoch with strong vegetation activity. There was no significant difference between the GSVIs of the 1980s and 2000s. The above finding suggests a slight fluctuation pattern between the three decades.

**Fig 6:**
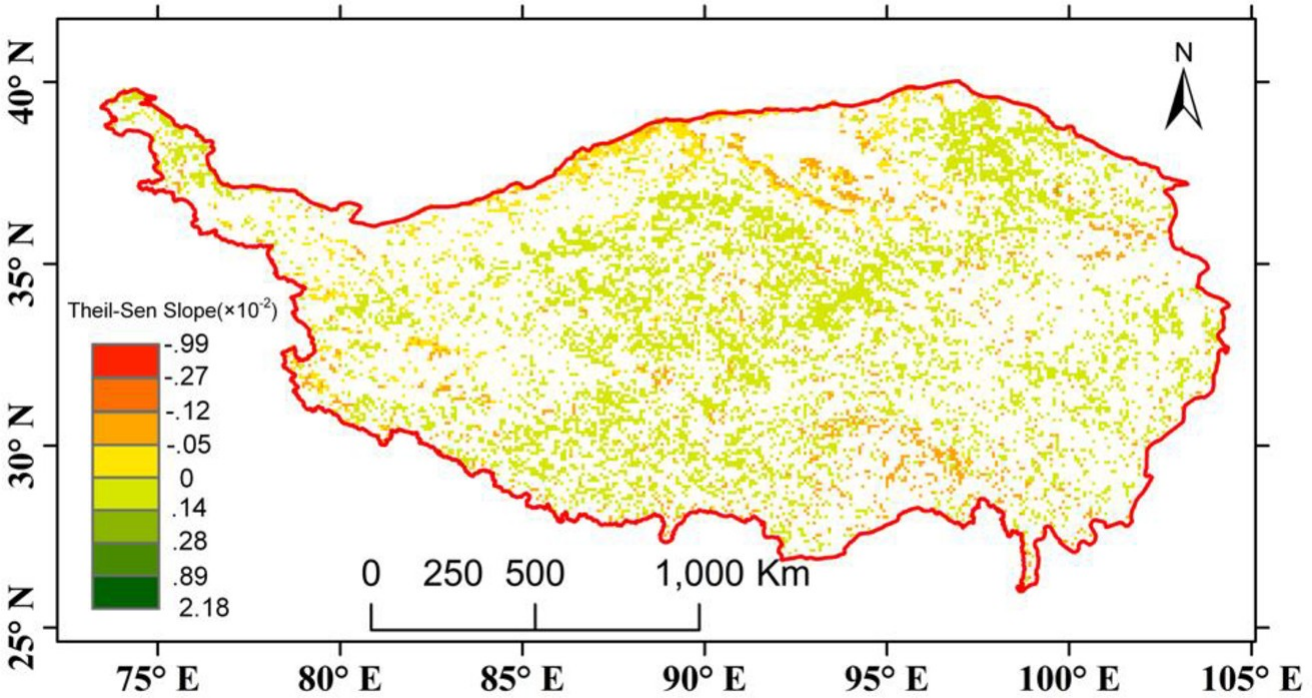
Spatial pattern of the Theil-Sen slope of the GSVI (only pixels with high MK significance (p < 0.05) are displayed). Red color represents highly negative trend, while green color represents highly positive trend.

Here, a total of 82% (9572) of all 8-km pixels of the TP showed significant trends for the Theil-Sen slope, of which 72.5% (6941) were positive and 27.5% (2631) were negative (Fig 6). These results suggested pronounced greening in the central region of the TP, most of which is alpine grassland. Although there was an overall greening trend of the vegetation throughout the TP, the trend did not have a very large magnitude. This trait can be identified from the fact that most of the pixels showing a positive trend fell in the slope interval 0–0.14. With respect to the spatial distribution of the negative pixels, there were some sparsely distributed pixels in the central regions. The southern belt of the Qaidam basin is a hotspot zone that aggregates a considerable number of negative pixels. In the central-southern area (near 95°E, 30°N), there are some regions with vegetation that showed a browning trend.

### Change points in the GSVI time series

**Fig 7:**
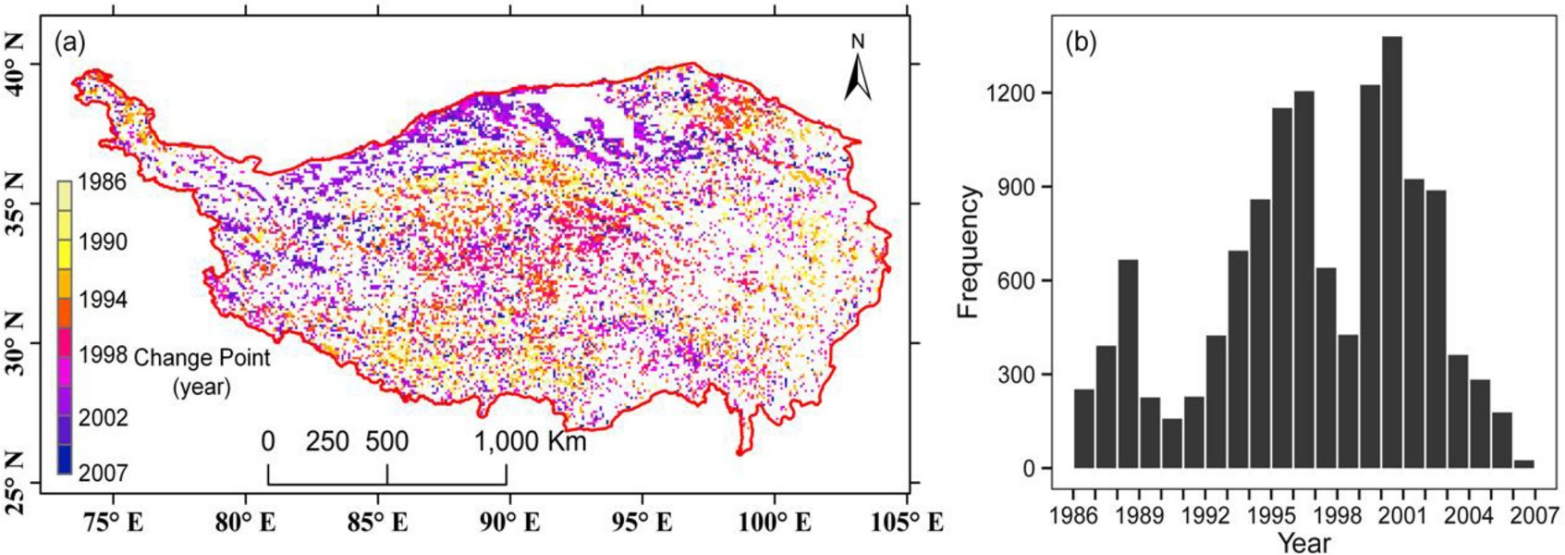
Spatial distribution of the change points (a) and their statistical histograms (b).

In addition to the monotonous trend test of the Theil-Sen slopes, we also extracted change time points for the dynamics of the vegetation. Change point analysis can capture the abrupt breaking point in the time series for each pixel. From the histogram of breakpoints, we found three peaks, which were approximately centered around 1988, 1997 and 2001 (Fig 7). The last two peaks were shaped by a gap from 1997–1998. From a spatial perspective, since the trend test above indicated an overall greening characteristic on the TP, there were a considerable number of pixels with change points. The most significant changes occurred widespread in the arid and semiarid regions on the northern part of the TP.

It should be mentioned that the change points in the GSVI time series could not be mainly attributable to climate or environmental changes. This situation may be induced by the difference in AVHRR sensors used by the GIMMS project. The sensor changes occurred in 1985, 1988, 1994, 1995, 2000 and 2004 [39,74]. Judging from the spatial variation in the change points, the sensor changes were not the dominant factor causing the trend to change. If this was the case, most significant change points would have occurred in similar years, resulting in a spatially homogeneous pattern of change points. However, according to our findings (Fig 7), the change points were distributed differently throughout the TP region. Previous studies also indicated that tendency analysis on GIMMS NDVI data was not affected by the sensor changes [75].

### Combined spatiotemporal variations

**Fig 8:**
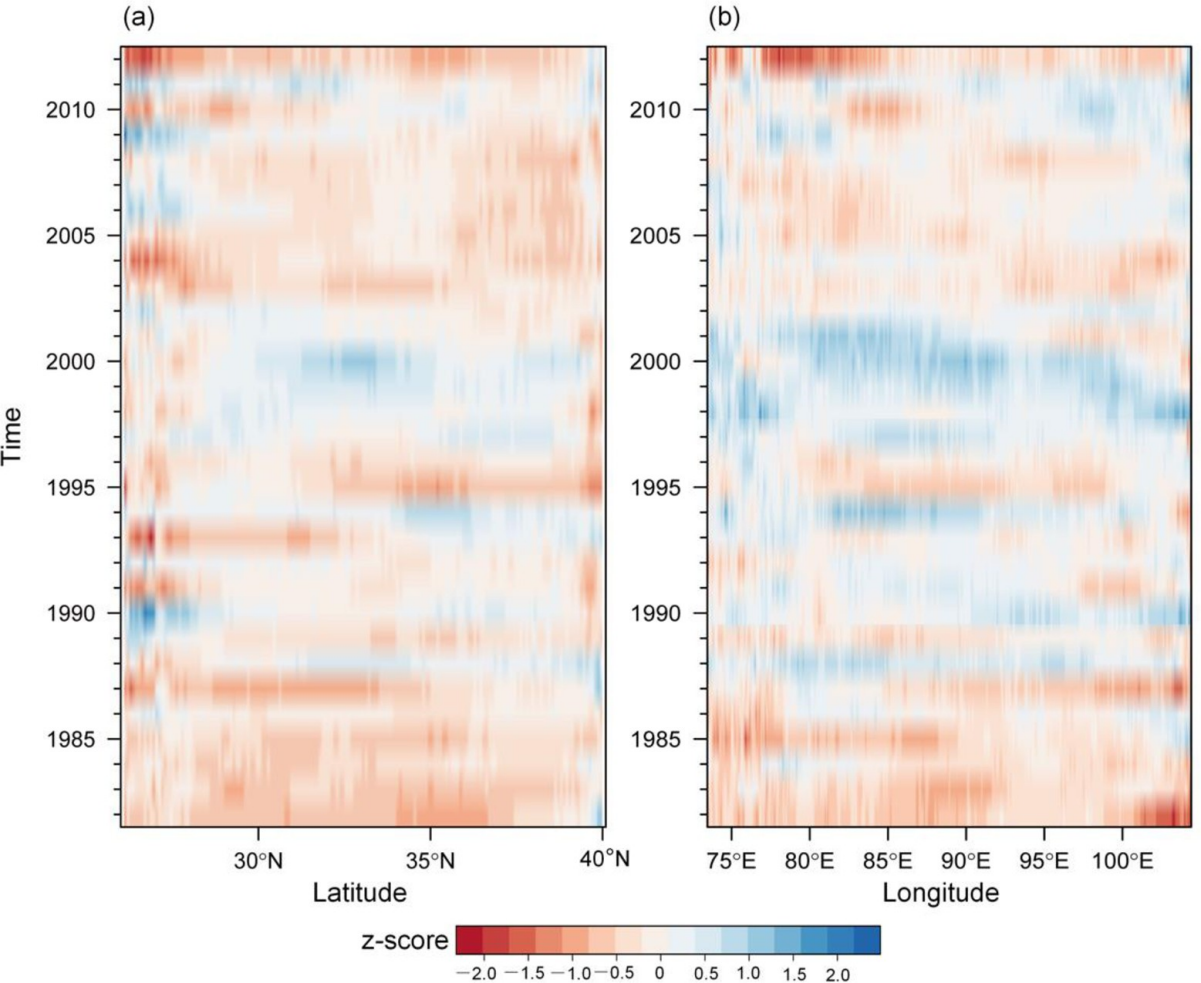
Hovmöller diagrams showing the spatiotemporal evolution of the GSVI standardized anomaly from January 1982 to December 2012. (a) Time-latitude distribution; (b) time-longitude distribution.

The standardized anomaly (z-score) of the GSVI was calculated to represent the departure condition of the vegetation greenness, which has been previously used to measure vegetation dynamics. For the annual GSVI, the z-score was computed by dividing anomalies by the standard deviation. Because the influences of dispersion have been removed, the z-score generally provided more information about the magnitude of the anomalies. Here, the Hovmöller diagrams were drawn through the vegetation z-scores to create time-latitude and time-longitude sections to examine the variation in the vegetation in the spatial and temporal dimensions. Fig 8 represents the standardized departure patterns, clearly revealing the extent and persistence of the greening and browning years. In the time-latitude section (Fig 8A), at low latitudes (approximately 25°N), there were three main time periods that showed greening (blue regions in Fig 8), which occured from 1989–1990, 2006–2007, and 2008–2009. Around the period from 1997–2000, the transitionary zones over the TP (approximately 27°N to 40°N) exhibited a pronounced area of positive z-scores. The spatially continuous greening pattern reflected that the vegetation was growing in a suitable environment. Meanwhile, there were some other very narrow greening belts that ran from the lower to higher latitudes that took place around 1988, 1994 and 2011. The browning pattern was significantly dominant in the time-latitude section. The spatiotemporal pattern in the time-longitude section is similar to that in the time-latitude section but clearly reveals a larger extent of greening, especially in the horizontal transection around the year 2000. Combining the two Hovmöller diagrams, we found that the central TP region was characterized by optimized vegetation conditions around the late 1990s and early 2000, followed by a browning period from 2003 to 2008.

**Fig 9:**
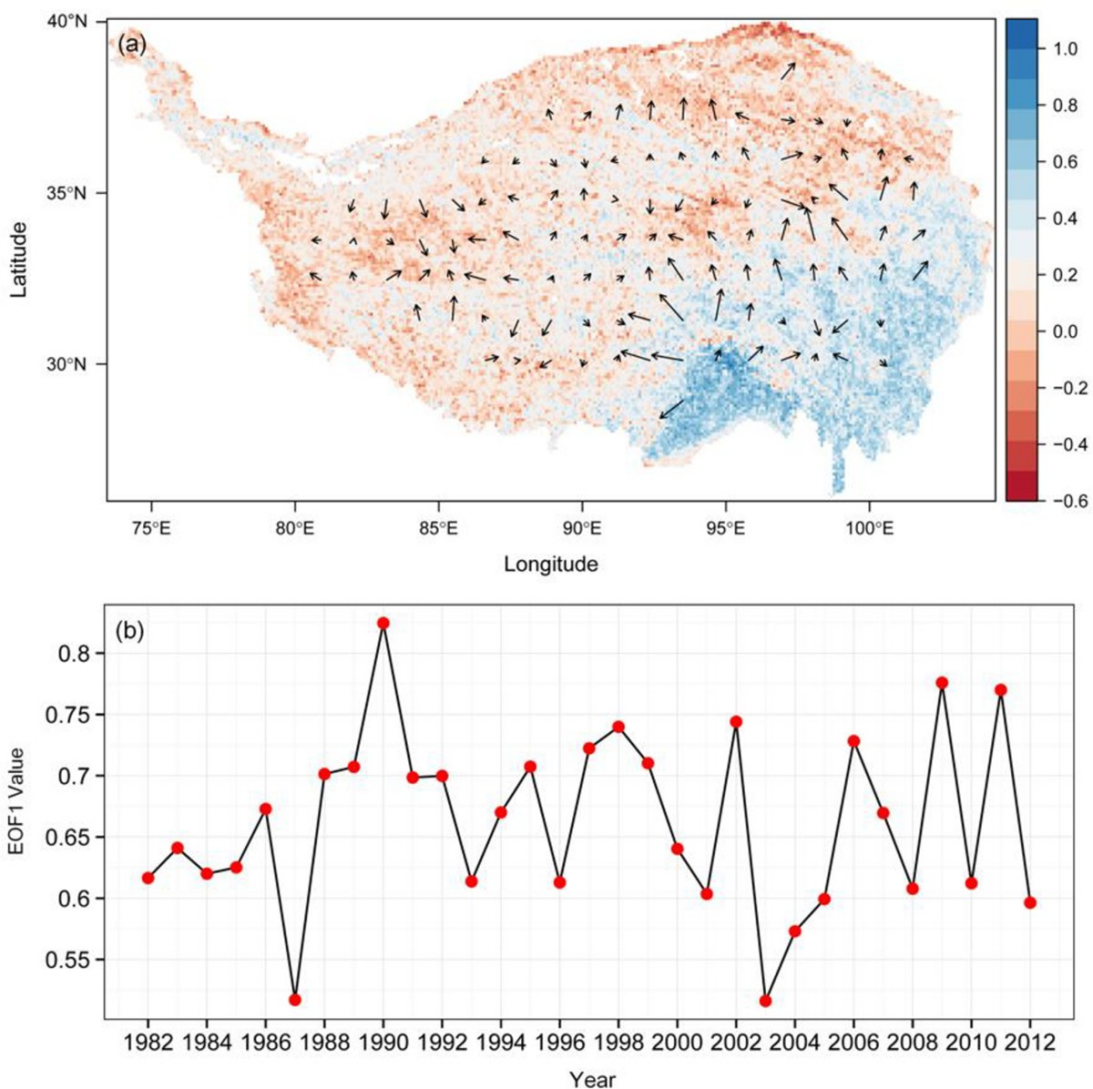
Spatial pattern of the first Empirical Orthogonal Function (EOF1) for the GSVI and the corresponding vector field (black arrows) (a), the time series of EOF1 (b). Color in upper figure indicates the capability of EOF1 in explaining the variability of NDVI time series.

Another spatiotemporal analysis method, EOF, was employed to examine the vegetation variability on the TP. In the EOF analysis, the significant EOFs represented stable patterns in a dataset, and the first few EOFs that explain the largest fraction of variance are usually selected. In this study, we chose the first ten EOF modes to decompose the raster stack after several adjustments for the output parameters. The first leading mode accounted for approximately 98% of all information from the time series of NDVI images (Fig 9A), along with the subsequent modes that explained much less of the variance. Thus, it was not surprising that the spatial structure of the first EOF resembled the surface topography of the plateau. From a spatial perspective, the first EOF followed a southeast-northwest gradient, transecting the area from the warm-humid southeast to the cold-dry northwest. The vector field also provides consistent insight, with a considerable number of black arrows pointing from the blue area in the southeast to the red area in the northwest. In comparison with the multiyear mean GSVI (Fig 3A), the first EOF also represented a similar spatial pattern, reflecting its capability to capture the major information of the long-term vegetation dynamics. From a temporal perspective (Fig 9B), the GSVI time series exhibited a pronounced fluctuation. There were two apparent troughs that occurred in 1987 and 2003, with an abrupt peak in 1990, which were also found in the overall spatial trend (Fig 3B). The fluctuation characteristic was also similar to that in Fig 3B, which generally showed an increasing trend in the 1980s and a significant interannual variation of the vegetation in the subsequent years.

### Vegetation dynamics at the ecoregion level

**Fig 10:**
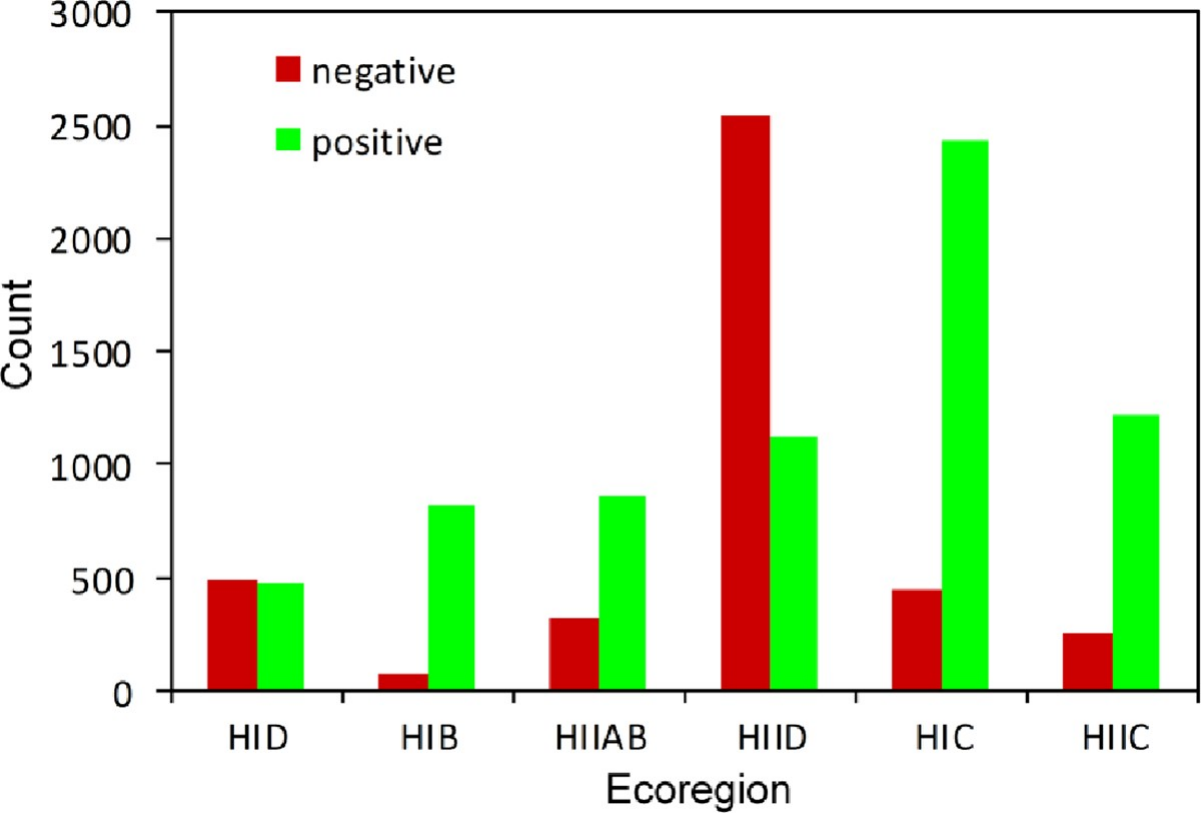
Pixel-level statistical results of GSVI change trends for the different ecoregions.

According to the previous analysis of the Theil-Sen slope for each pixel, we calculated the long-term trend for the pixels in each ecoregion and classified them into two classes (negative and positive). As shown in the histogram (Fig 10), it was apparent that the distribution of the vegetation change statuses was variable among the different ecoregions. The vegetation growth tendency in most ecoregions was controlled by a positive trend (green bars), with HID and HIID representing the opposite trend. In the HIC and HIIC ecoregions, there were more positive pixels than negative pixels, revealing a long-term trend of increasing greenness of the vegetation. In contrast, HIID was significantly dominated by the negative trend, which had nearly twice the influence of the positive trend. In addition, the HIC had the largest area (number of pixels) showing positive trends, while the largest area with a decreasing growth trend occurred in the HIID region. In the frigid arid zone (HID), the negative area nearly equaled the positive area. These results reflected that the drought regions tended to continuously lose plant coverage over the three decades. The semiarid regions became greener. The humid and semihumid regions still maintained greening tendencies with some decrease.

**Fig 11:**
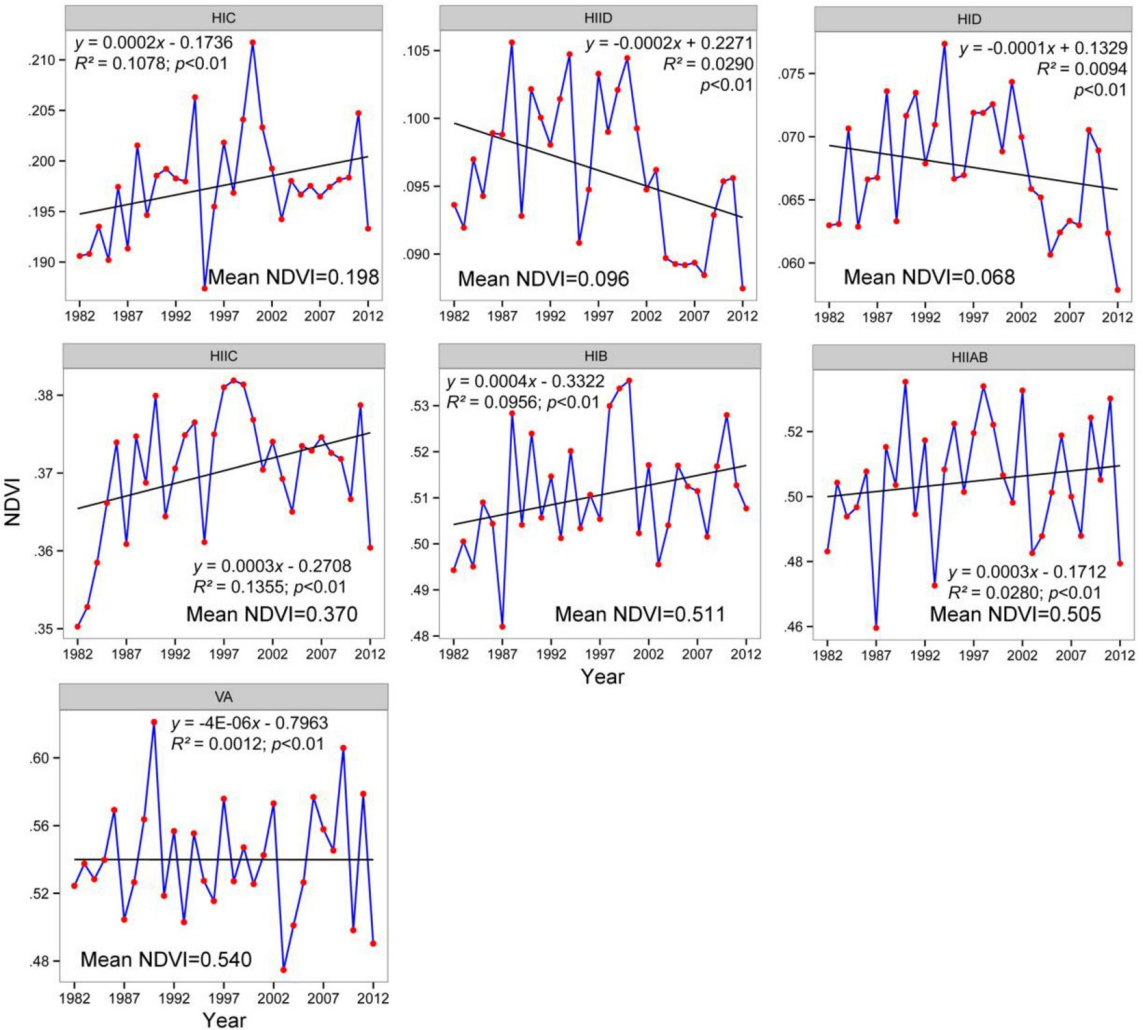
Interannual dynamics and trends of the GSVI in each ecoregion. The multiyear averaged GSVI value is labeled on the plot panel.

The time series of interannual GSVI for each ecoregion is drawn in Fig 11. To examine the long-term trends of the vegetation, we performed a simple linear trend analysis for these time series. In this case, all the ecoregions experienced a strong interannual variation in vegetation cover over the three decades. Generally, these long-term trends for the ecoregions could be aggregated into two classes: decreasing and increasing trends. It is significant that the arid regions (i.e., HID, HIID) presented a decreasing trend and varied slightly. In particular, the temperate arid zone (HIID) showed the strongest decreasing trend, indicating the serious degradation of the vegetation in the northern part of the TP. Although both HID and HIID showed decreasing vegetation conditions, their fluctuation ranges for the annual growing season NDVI were very different (0.058–0.078 VS. 0.087–0.106). The lowest HIID value was larger than even the maximum HID value, which can be attributed to the temperature characteristics of the two ecoregions. Their corresponding 31-year averaged growing season NDVI also confirmed this difference (0.096 VS. 0.0676).

For the semiarid regions, both HIC and HIIC experienced significant increases in GSVI, while HIIC experienced more rapid greening. Similar to the difference between HID and HIID, HIIC had a much higher multiyear mean NDVI than HIC. By carefully observing the time profile in Fig 11, except for the VA ecoregion, we can see that the GSVI in the first years of the timeseries demonstrated a significant ascending trend, which was especially apparent for the arid and semiarid regions (HIC, HIIC, HID, HIID). The slopes for the entire time period and the first decade were calculated (Table 2). Obviously, for each ecoregion, the magnitude of the slope for the first decade remarkably surpassed that for the thirty-one-year period, and all the ecoregions exhibited positive increasing trends. This finding was consistent with the result derived from the intercomparison of the three decades of GSVI for the entire TP area (Fig 5). Both showed that the 1980s was a good growing time for vegetation throughout the TP. For the HID and HIID regions, even though they had negative tendencies over the three decades, both showed an evident positive and similar slope (0.0009)in the first decade. This kind of reversal with respect to the slope was much greater for the VA ecoregion (-4E-06 VS. 0.0037).

**Table 2:** Spatially averaged GSVI and coefficients of variation for seven ecoregions.

	HIC	HIID	HID	HIIC	HIB	HIIAB	VA
30-yr Mean NDVI	0.198	0.096	0.0676	0.370	0.511	0.505	0.540
Slope (1982–2012)	0.0002	-0.0002	-0.0001	0.0003	0.0004	0.0003	-4E-06
Slope (1982–1991)	0.001	0.0009	0.0009	0.0023	0.0021	0.0023	0.0037
Coefficient of variation (%)	2.61	5.48	6.93	2.18	2.45	3.72	6.18

In addition to the growth trend of the vegetation over the three decades throughout the TP, we also calculated the coefficient of variation (CV) to quantify the interannual variations in the ecoregion-averaged GSVI. This quantitative result was similar to the fluctuations observed in the plots (Fig 11). The greatest interannual variation occurred in the arid regions in the northern TP and VA regions in the south, as indicated by their CVs of 6.93 and 6.18 (Table 2). Meanwhile, the semiarid regions in the HIC and HIIC show the same levels of interannual variation, with HIC having a slightly higher value. This finding suggested that the vegetation in the northern regions of the TP, especially the northern areas of the Kunlun Mountains and Qaidam basin, varied the most over the three decades. In the southeastern ecoregions, the variation decreased from the southern HIIAB to the northern HIB. In total, the central TP, which consists of HIC, HIIC and HIB, presented a relatively lower level of vegetation variation, which indirectly reflects the stable development conditions of the highland grasslands. The drought and VA zones exhibited more obvious variations than the other ecoregions, as confirmed by the coefficient of variation (Table 2 and Fig 4).

### Future vegetation trends after 2012

**Fig 12:**
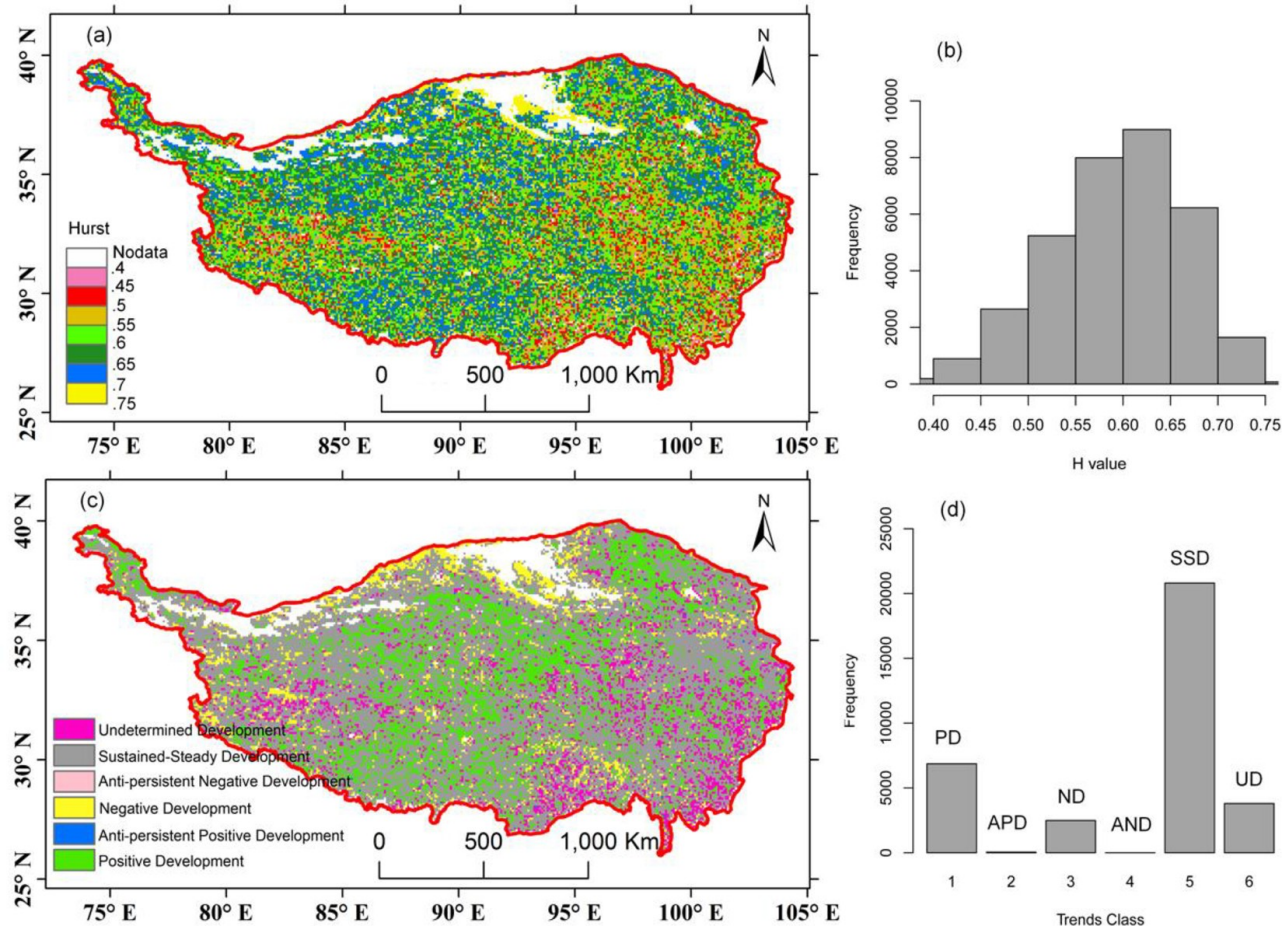
Spatial pattern of the Hurst exponent (a) and future vegetation trends (c) derived from GIMMS NDVI data (1982–2012). (b) and (d) are the corresponding statistical distributions for (a) and (c).

For the entire TP, the spatially averaged H value was 0.59, which reflected an overall persistent vegetation trend at a medium level. From Fig 12, we can see that most of the vegetated region had a time series with persistent behavior (30165 pixels; 88.7% of plants covered area). A total of 3851 pixels (11.3% of plants covered area) have a time series with anti-persistent behavior. Areas with negative H values are mainly located in the southeastern part of the study area, and some patches are located in the southern and western parts. The regions with the highest persistent vegetation trends were distributed in the northern arid area (yellow color in Fig 12C). In particular, the mean H values in the majority of the ecoregions were above 0.5, but these values had a small range (0.541–0.626). VA6 had a mean H value (0.54), followed by HIIAB1 (0.56), HIB1 (0.57), HIID3 (0.592), HIIC1 (0.593), HIC1 (0.596), HIIC2 (0.596), HID1 (0.604), HIID2 (0.617) and HIID1 (0.626). Only VA5 had an H value of 0.498, which was very close to 0.5. This finding indicated that, at the regional scale, the vegetation in arid and semiarid areas had relatively more persistent trends than those in humid areas.

The GSVI Theil-Sen slopes and Hurst exponent were combined to classify the vegetation future trends into six classes (Fig 12C). Most of the vegetated areas on the TP were projected to have stable and steady trends in the future (SSD; 61.2% of the vegetated area), indicating that they had nonsignificant development trends and H values above 0.5. The areas with negative development (ND; 7.3% of the vegetated area) of vegetation were mainly located in the northern part of the Qaidam basin margin (Fig 12C). This vegetation showed a pronounced negative future trend that can be attributed to the low precipitation in the arid areas. Approximately 20.1% of the areas were covered with vegetation, indicating positive future trends (PD) in these areas, which were distributed in the northeastern, central and southwestern parts of the TP. There were rare pixels where the vegetation displayed anti-persistent negative trends (AND). In the eastern, southeastern and western regions, which are mainly the HIB1, HIIAB1 and VA ecoregions, there were a considerable number of pixels with undetermined future trends (UD; 11.2% of the vegetated area). The areas with anti-persistent positive trends only accounted for 0.15% of the plant-covered areas.

### Relationship of vegetation with temperature and precipitation

**F**rom Fig 2, temperature in the majority of the TP presents a warming trend, with the southwestern part having a significant rise of temperature. But northwestern part has a pronounced decline of temperature. Precipitation in the northern and western TP tends to increase slightly, but decreases in the southeastern and southwestern parts.

**Fig 13:**
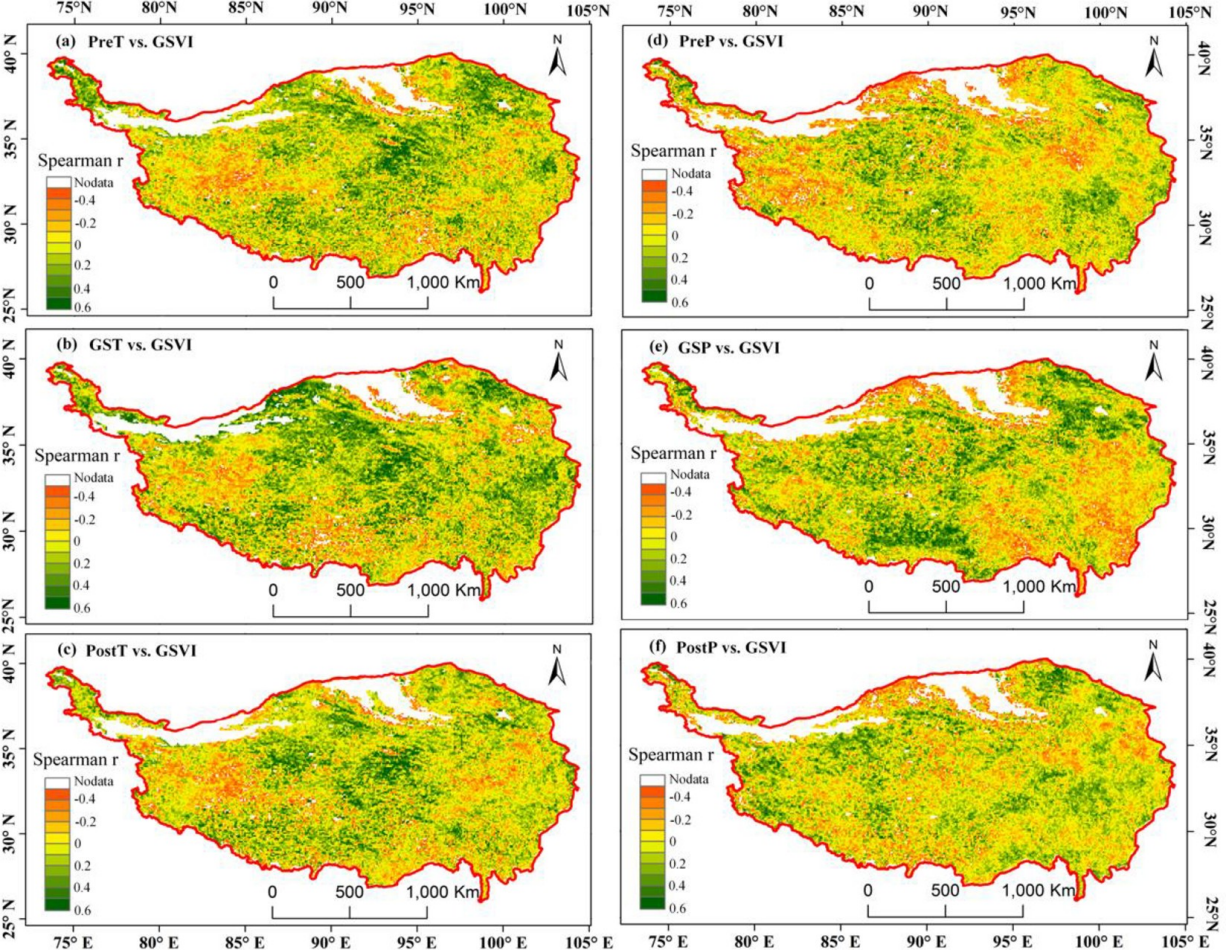
Spearman correlations between the GSVI and PreT(a), GST(b), PostT(c), PreP(d), GSP(e), and PostP(f).

As shown in Table 3, the pre-season temperature (PreT) positively correlated with the GSVI in a large part of the eastern, central and northern TP (60.9% of vegetated area). Negative correlations mainly occurred in the southwestern and western regions of the TP (Fig 13A). In 63.89% of the vegetated TP area, the growing season temperature (GST) exhibited a positive relationship with the GSVI, except in the western, southern and northeastern parts, which showed negative relationships. The spatial pattern of correlation between the post-season temperature (PostT) and GSVI was similar to that between the PreT and GSVI, which had a relatively small correlation. For pre-season precipitation (PreP) vs. GSVI (Fig 13D), the northern, eastern and western portions of the TP exhibited negative relationships. In the eastern and southeastern parts and northern TP edge, the growing season precipitation (GSP) vs. GSVI displayed negative correlations. Compared to the GSP, the post-season precipitation (PostP) has a relatively smaller magnitude of correlation with the GSVI. Their positive relationships were sporadically distributed mainly in the western, northeastern and eastern parts of the TP.

**Table 3:** Pixel statistics for the relationship of the vegetation with the temperature and precipitation.

	PreT vs. GSVI	GST vs. GSVI	PostT vs. GSVI	PreP vs. GSVI	GSP vs. GSVI	PostP vs. GSVI
Positive (+)	60.9%	63.89%	58.5%	51.2%	55.5%	55.8%
Negative (-)	39.1%	36.11%	41.5%	48.8%	44.5%	44.2%

**Fig 14:**
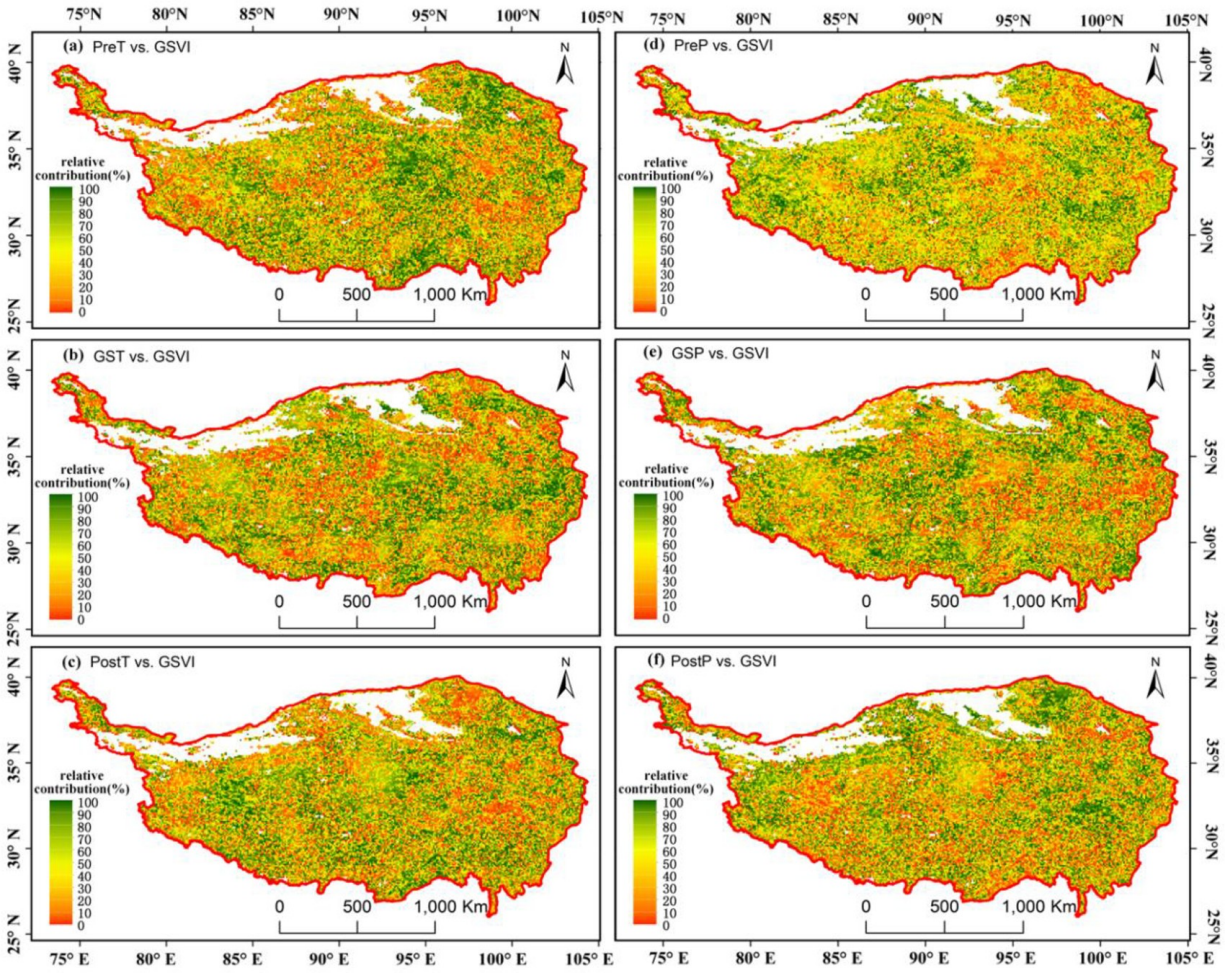
Relative contribution of the PreT(a), GST(b), PostT(c), PreP(d), GSP(e), and PostP(f) to the GSVI.

The relative importance method is an effective tool for detecting the contributions of temperature and precipitation to the regulation of the changes in vegetation. For the geographical distribution, the temperature contribution to the GSVI presented a contradictory pattern with precipitation, both during the preseason, growing season and postseason. For instance, it was more apparent in the central TP where the preseason temperatures played a dominant role (green area denoting high value of relative importance), while the preseason precipitation had a lower contribution (red area) (Fig 14A and 14D). The temperature contributions in the three periods demonstrated a similar spatial pattern, but the magnitude decreased sequentially from preseason to postseason. There was a nearly identical pattern in the precipitation contribution, although the growing season rainfall enhanced the greenness in northeastern arid areas.

## Discussion

### Potential drivers for vegetation dynamics

This study showed that the vegetation has experienced a greening trend over the entire TP, which is consistent with recent findings [31,67]. In the literature on the TP, the vegetation greenness and key drivers have been widely reported, especially the impacts of the climatic factors on vegetation. The rate of temperature increase on the TP was shown to be faster than that in the Northern Hemisphere [76], which may enhance the vegetation activity and lengthen the growing season. A previous study found that there was a strong relationship between the NDVI and rainfall in the meadows and grasslands with medium vegetation cover on the TP [26]. In this study, although precipitation was slightly associated with vegetation greenness, it had a lower impact than those of temperature. This may be attributed to the scarcity and the unequally spatial distribution of the precipitation. Whereas vegetation presenting positive trend mainly occurred in the regions with relatively moist condition, such as in meadow areas.

The relatively small changing trend of the vegetation from the late 1990s to 2000s was another finding in this paper, which may be associated with the global warming hiatus during 1998–2012 [73]. This reversal of global warming may result in some changes in vegetation growth. In the statistical distribution of the change points, the year 1998 was an apparent time point that fell in the trough of a wave after which there was a peak consisting of numerous change points (Fig 7), which suggests the response of vegetation to global warming.

The shifts and dynamics in the vegetation time series may not only be attributed to the breaking point of the natural factors but also could be induced by anthropogenic perturbations, such as grassland management. The change direction in the turning point could be classified into several types, such as greening to browning, browning to greening, greening to higher (lower) greening, and browning to higher (lower) browning. On the northern TP, especially in the alpine grassland, the persistent overgrazing has exceeded the capacity of the available pastures. The local and central governments have implemented a grazing exclusion policy since the 1990s. As a result, fenced grassland patches have been built to exclude grazing activities [77]. These management activities may promote ecosystem conditions, such as restoration of degraded natural alpine grasslands. The finding of change points could partially be induced by these human activities. However, according to previous studies, the grassland fencing policy is not a key factor in controlling vegetation changes [77,78]. In that case, natural forces, such as environmental factors or elevation conditions at the local, regional or global scales, would dominate the shifts in vegetation. In fact, stock farming is not a traditional form of grazing, but nomadic herding is a lifestyle that has a long history for the people of the TP. The natural vegetation is thought to maintain a dynamic balance between plant production and livestock count.

### Limitations of the study

It is noteworthy that this study focused on the long-term dynamics and variability of the growing season vegetation on the TP. To obtain a comprehensive understanding of the natural vegetation changes on the TP, more factors and model simulations, such as those of larger-scale climate change, atmospheric circulation, and detailed human activities, should be considered in future studies. In addition, the GIMMS NDVI3g (V0) data have a spatial resolution of 8 km on the ground, which limits the capability in depicting vegetation cover at a fine spatial resolution. The emergence of satellite images with high spatiotemporal resolutions, such as Landsat 8 and Sentinel-2, will provide pronounced potential in enhancing vegetation monitoring in the future.

## Conclusion

Using GIMMS NDVI3g data, this study assessed the long-term trends and variability of the growing season vegetation on the TP (1982–2012). The temporal and spatial changes in the GSVI were assessed using the Theil-Sen slope, change point detection, variation and EOF methods at the pixel and regional scales. The growing season vegetation throughout the entire study area experienced greening conditions, especially in sub-frigid semi-arid regions, but with small magnitudes and complex spatiotemporal dynamics. Vegetation during 1990s shows more obvious growing activities and change points. Most of the vegetation on the TP may maintain the persistent growing trend in the future.
